# Pregnancy stress in women at high risk of preeclampsia with their anxiety, depression, self-management capacity: a cross-sectional study

**DOI:** 10.3389/fpsyg.2025.1537858

**Published:** 2025-05-21

**Authors:** Xing Cong, Jinmei Wang, Liu Yang, Lingling Cui, Yurong Hua, Ping Gong

**Affiliations:** ^1^Department of Obstetrics, Affiliated Hospital of Jiangnan University, Wuxi, Jiangsu, China; ^2^School of Wuxi Medical College, Jiangnan University, Wuxi, Jiangsu, China; ^3^Department of Obstetrics, Women’s Hospital of Jiangnan University, Wuxi, Jiangsu, China

**Keywords:** preeclampsia, pregnancy stress, anxiety, depression, self-management capacity

## Abstract

**Introduction:**

Many studies suggest that psychological factors are intrinsically connected to the onset of preeclampsia. However, there are no relevant surveys on the psychological situation of this population. The aims of our study were to investigating the causes and prevalence of pregnancy stress in individuals at high risk of preeclampsia; exploring the correlation between pregnancy stress and anxiety, depression, and self-management capacity in this group. Our study provided evidence for the development of effective clinical management strategies and related psychological care for women at high risk of preeclampsia.

**Methods:**

A cross-sectional survey was conducted on women at high risk of preeclampsia who came to Jiangnan University Hospital’s obstetrics outpatient clinic for antenatal care. Sociodemographic and obstetric-related characteristics, Pregnancy Stress Rating Scale (PSRS), Self-rating anxiety scale (SAS), Self-rating depression scale (SDS), Self-rating Questionnaire of Healthcare Management for Pregnancy (SQHMP) were included in this study. Data analysis covered descriptive statistics, univariate analysis, Spearman’s rank correlation, and multiple linear regression analysis.

**Results:**

A total of 138 pregnant women at high risk of preeclampsia were enrolled in the study. Univariate analysis showed significant relationships between intergenerational relations (with mothers-in-law), pregnancy intention, and desired mode of delivery among pregnancy stress (*p* < 0.001). Median (IQR) scores were: PSRS 0.283 (0.133, 0.542), SAS 38.750 (32.500, 45.000), SDS 41.250 (33.750, 50.000), SQHMP 43.000 (35.000, 53.250). The score of Spearman’s rank correlation showed that pregnancy stress was positively correlated with anxiety, depression, and self-management capacity, respectively (r = 0.465, *p* < 0.001), (r = 0.437, *p* < 0.001), and (r = 0.585, *p* < 0.001). Multiple linear regression analysis showed that desired mode of delivery, anxiety, and self-management capacity were the main predictors of pregnancy stress.

**Conclusion:**

The findings emphasize the need to focus on pregnancy stress in women at high risk of preeclampsia, especially those have presented higher levels of anxiety, depression, and self-management capacity. Based on these variables, healthcare professionals should increase screening for mental health in pregnant women at high risk of preeclampsia as well as provide additional psychological care.

## 1 Introduction

Preeclampsia is a serious pregnancy-specific condition characterized by new-onset hypertension and proteinuria after 20 weeks of gestation (Rana et al., 2020). It is estimated to affect approximately 2–8% of pregnancies worldwide (Gatford et al., 2020). In addition to raising the maternal risk of eclampsia, HELLP syndrome, and multi-organ dysfunction, preeclampsia can cause fetal growth restriction (FGR), preterm birth, and intrauterine fetal demise (Ives et al., 2020). However, to date, no single pathophysiology can have been able to explain the emergence of preeclampsia (Phipps et al., 2019). As the traditional medical care paradigm shifts, there is a growing emphasis on the role of maternal psychosocial factors in the prevention, treatment and regression of the disease (Freiberger et al., 2022). Previous research suggests that psychological factors are intrinsically connected to the onset of preeclampsia (Krishnamurti et al., 2019; Traylor et al., 2020).

Along with the physiological changes, pregnant women also face alterations in their social, economic, familial, and personal roles. This makes them particularly vulnerable to psychological changes and mood swings, ultimately leading to pregnancy stress (Wang et al., 2023; Heberlein et al., 2016; Niela-Vilen et al., 2023). Literature suggested that almost 80% of pregnant women both at home and abroad experienced varying levels of pregnancy stress (Na et al., 2022). Research on pregnancy stress in China has developed relatively later compared with Western countries, with significant regional variations in stress levels among pregnant women (Caiyun et al., 2020). As an economically developed city in the Yangtze River Delta region, Wuxi exposes women of childbearing age to characteristic urban stressors including work–family conflicts and high living costs (Shuxiu, 2021). Notably, studies have reported that the prevalence of preeclampsia in metropolitan areas in southern China ranges from 4.4 to 5.57%, which is at a high level both at home and abroad (Gatford et al., 2020; Caixia et al., 2019). Pregnant women are prone to anxiety and depression due to the numerous stressors they encounter (Delagneau et al., 2023). A survey of primiparous women showed that higher levels of psychological stress were associated with more anxiety and depression during pregnancy (Hang et al., 2017). Pregnant complications such as habitual abortion, gestational hypertension, and preterm labor may become more common if negative emotions persist (Bernard et al., 2019). In a meta-analysis by Shay et al. (2020), the results revealed that there was a 40% increased risk of preeclampsia among women who suffered adverse mood at any time during pregnancy. Another research reported that both pregnancy stress and risk factors for preeclampsia accounted for developing preeclampsia in a synergistic manner (Giurgescu et al., 2015; Yu et al., 2013). Especially in recent years, neuropsychoimmunological mechanisms explaining the causal relationship between stress and preeclampsia are becoming a topic of active research (Buglione-Corbett et al., 2018). Psychological stress during pregnancy induces sympathetic arousal and activation of the hypothalamic–pituitary–adrenal axis, which may promote endothelial dysfunction and increased inflammatory activity (László et al., 2013), finally leading to the occurrence of preeclampsia.

Preeclampsia management remains clinically challenging. Current interventions focus on intensive monitoring (blood pressure, proteinuria, organ function) and symptomatic treatment to prolong gestation safely (Amaral et al., 2017). Definitive treatment requires pregnancy termination, often necessitating iatrogenic preterm delivery when maternal-fetal risks escalate (Dimitriadis et al., 2023; Smith et al., 2020). As modifiable risk factors, psychosocial stressors (especially pregnancy stress, anxiety, and depression) and self-management capacity warrant further investigation regarding their association with preeclampsia development (Ngene and Moodley, 2024; Bosquet Enlow et al., 2021). In the past, more attentions have been paid to women who have already experienced preeclampsia than to those who are more likely to get preeclampsia. Moreover, there has not been much research done on quantifying and evaluating the psychological conditions associated with those at high risk of preeclampsia (Rahnemaei et al., 2020). Behavioral interventions during pregnancy may affect maternal stress and mental health (Bosquet Enlow et al., 2021). Several studies have highlighted the importance of personal self-management for improving preeclampsia awareness and some pregnancy outcomes in pregnant women at high risk for preeclampsia (Alnuaimi et al., 2020; Moulaei et al., 2021; Endeshaw et al., 2015). However, whether the positive effects caused by self-management are related to pregnancy stress, anxiety, and depression remains to be explored.

In conclusion, the aims of our study were to investigating the causes and prevalence of pregnancy stress in individuals at high risk of preeclampsia; exploring the correlation between pregnancy stress and anxiety, depression, and self-management capacity in this group. Having a thorough understanding of these factors supports the creation of clinical management plans and related psychological services. This encourages multifaceted prevention of unfavorable pregnancy outcomes.

## 2 Materials and methods

### 2.1 Study setting and participants

**Figure 1:**
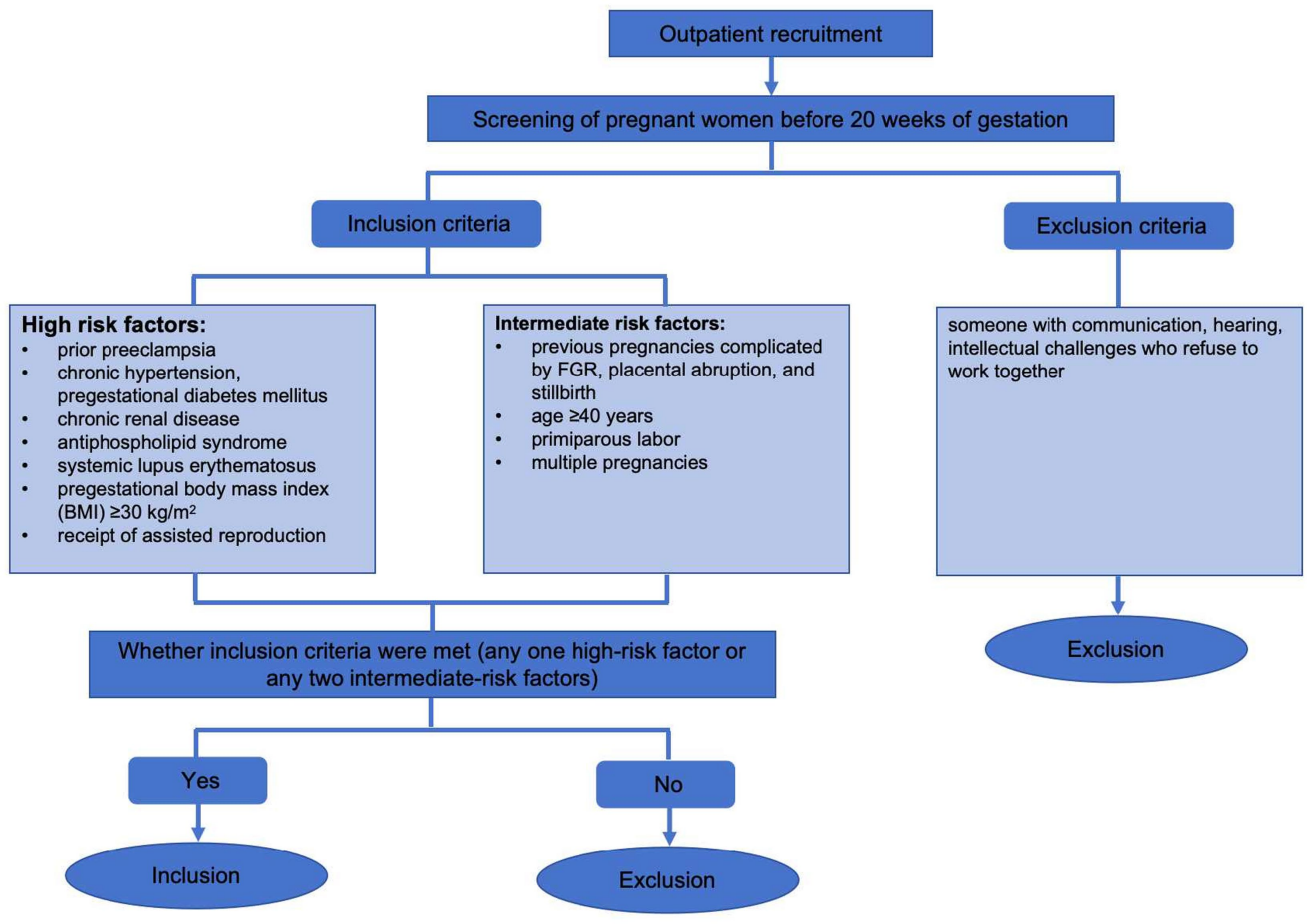
Participant recruitment flowchart.

A cross-sectional relational design was used in this study. From May 2023 to August 2024, we looked into the women at high risk of preeclampsia before 20 weeks of pregnancy and came to obstetrical outpatient clinic affiliated Hospital of Jiangnan University in Wuxi City, Jiangsu Province, China, for antenatal care. The inclusion criteria for women were: any one high-risk factor or any two intermediate-risk factors (Yao Tang and Li, 2022). High risk factors: prior preeclampsia, chronic hypertension, pregestational diabetes mellitus, chronic renal disease, antiphospholipid syndrome, systemic lupus erythematosus, pregestational body mass index (BMI) ≥ 30 kg/m^2^, receipt of assisted reproduction; intermediate risk factors: previous pregnancies complicated by FGR, placental abruption, stillbirth, and multiple pregnancies, age ≥ 40 years, primiparous labor. Exclusion criteria: someone with communication, hearing, and intellectual challenges who refuse to work together. Based on the parameters of previous related studies (Tuxunnjiang et al., 2022) r = 0.278, 1-*β* = 0.9, the sample size was calculated to be 131 pregnant women using G power 3.1 software, which was further expanded by 5% considering invalid questionnaires at the time of sample retrieval. The estimation showed that the inclusion of at least 138 pregnant women at high risk of preeclampsia fulfilled this investigation. The detailed recruitment process was shown in Figure 1.

### 2.2 Ethical consideration

This study was approved by the Ethics Committee of the Affiliated Hospital of Jiangnan University (LS2024302) and the informed consent of the pregnant women has been obtained. The study was conducted in accordance with the guidelines of the Declaration of Helsinki.

### 2.3 Data collection

Before data collection, all researchers received standardized training. Using established protocols, they explained the study and obtained informed consent before conducting face-to-face interviews to ensure consistent questionnaire administration and interpretation. The data collection method was a combination of electronic medical record review and a paper questionnaire survey. Questionnaires were distributed and returned on the spot 150 questionnaires were handed out during the survey’s May to August 2024 period. Twelve of these were excluded from the analysis due to dropping out (Freiberger et al., 2022) and incomplete data (Traylor et al., 2020), leaving 138 questionnaires for final analysis.

### 2.4 Measures

#### 2.4.1 Sociodemographic and obstetric-related characteristics

Age, education, residence, employment status, monthly per capita household income (RMB, renminbi, Chinese yuan), marital relationships and intergenerational relations (with mothers-in-law), sleep situation, pregnancy intention, and desired mode of delivery were all gathered using a self-designed questionnaire. Others were collected through medical records.

#### 2.4.2 Pregnancy stress rating scale (PSRS)

The Pregnancy Stress Rating Scale (PSRS) is a self-reported assessment tool created in 1983 by Chen, a Chinese researcher from Taiwan. It gauges prenatal stress in expectant mothers (Chen, 2015). Four dimensions are represented by the scale: (a) parental role identification; (b) mother and child health and safety; (c) changes in body size and physical activity; and (d) additional stressors. The PSRS consists of 30 items, and the scale score = actual total pregnancy stress score/ number of all entries. Higher scores correspond to higher levels of pregnancy stress. With 0.000 for no stress, 0.001–1.000 for mild stress, 1.001–2.000 for moderate stress and 2.001–3.000 for severe stress (Tang et al., 2019). Prior research has demonstrated the scale’s validity in Chinese women (Cronbach’s *α* = 0.936–0.946) (Gao et al., 2023). The Cronbach’s alpha for the PSRS in this study wa*s* 0.939.

#### 2.4.3 Self-rating anxiety scale (SAS)

The 20-item Self-Rating Anxiety Scale (SAS) was compiled by American doctor Zung (1971). The SAS is a self-reported assessment tool. Its total score can be as low as 0 and as high as 100, with the standardized score being the sum of the scores on the 20 items multiplied by 1.25. Based on the Chinese SAS criteria, which represent the subjective emotions of those who are prone to anxiety, a cut-off point for anxiety was established at a total score of 50. As the score increases, the anxiety increases. The cut-offs for the SAS standard scores were defined as: <50, no anxiety; 50–59, minimal to mild anxiety; 60–69, moderate to marked anxiety, >70, severe anxiety (Zhou et al., 2022). The Cronbach’s alpha for the SAS in this study was 0.705.

#### 2.4.4 Self-rating depression scale (SDS)

The Self-rating depression scale (SDS) is a self-reported assessment tool. Its total score can be as low as 0 and as high as 100, with the standardized score being the sum of the scores on the 20 items multiplied by 1.25. Based on the Chinese SDS criteria, which represent the subjective emotions of those who are prone to depression, a cut-off point for depression was established at a total score of 50.Standard score is classified as: <50, no depression; 50–59, minimal to mild depression; 60–69, moderate to marked depression, >70, severe depression (Zhou et al., 2022). The Cronbach’s alpha for the SDS in this study was 0.848.

#### 2.4.5 Self-rating questionnaire of healthcare management for pregnancy (SQHMP)

In order to evaluate pregnant women’s self-management capacity, Jinzhi Li (2011) created the Self-rating Questionnaire of Healthcare Management for Pregnancy (SQHMP) in 2013, which is also relies on self-reported. Each entry is scored from 1 to 5 points, with a total score of 25–125 points, higher scores mean that pregnant women have better self-management capacity. Strong reliability and validity were demonstrated by the questionnaire’s internal consistency reliability coefficient of 0.93, retest reliability Pearson’s correlation value of 0.96, and content validity index (CVI) of 0.91 (Xiao et al., 2024). The Cronbach’s alpha for the SQHMP in this study was 0.888.

#### 2.4.6 Data analysis

SPSS 25.0 statistics software was used for statistical analysis after the gathered data was carefully checked and entered into Excel to create a database. In descriptive statistics, pregnant women are represented as n (%) in the general information. Since the data for the questionnaire score petition did not follow a normal distribution, we used median and interquartile spacing to represent them. The Mann–Whitney *U* test or the Kruskal-Wallis H test were employed in the univariate analysis of mothers’ stress when assessing the variations in pregnancy stress among obstetric and demographic characteristic variables. Since the data on pregnant stress, anxiety, depression and self-management skills during pregnancy in our sample did not conform to a normal distribution, the application of Spearman rank correlation analysis was considered. Multiple linear regression models were employed to determine factors that contribute to pregnancy stress in the end. All categorical variables included in the model were coded with appropriate dummy variables prior to inclusion in the model (Intergenerational relations: 1 = Cohesion, 2 = General, 3 = Occasional quarrel; Planned Pregnancy: 0 = Yes, 1 = No; Desired mode of delivery: 1 = Vaginal delivery, 2 = Cesarean delivery. SAS, SDS, and SQHM are substituted with the original value of the total score). Statistical significance was defined as *p* < 0.05, and all tests were two-sided.

## 3 Results

### 3.1 Demographics and clinical characteristics of obstetrics

Societal and obstetric attributes of the 138 individuals in the study population are shown in Table 1. All participants ranged in age from 18 to 43 years with a mean of (31.93 ± 5.10) years. For this pregnancy, the majority of women (68.1%) had a planned pregnancy. Table 1 displays the remaining obstetric clinical and sociodemographic features.

**Table 1:** Differences in sociodemographic and medical information variables according to pregnancy stress scores of participants (*N* = 138).

Characteristics	Case (*n*)	Percentage (%)	Pregnancy stress score, M (P_25_, P_75_)	Z/H	*P*
Age (years)				−1.253	0.210
<35	98	71.0	0.333 (0.133, 0.567)		
≥35	40	29.0	0.267 (0.100, 0.433)		
BMI (kg/m^2^)				0.442	0.931
<18.5	7	5.1	0.300 (0.033, 0.667)		
18.5–24.9	55	39.9	0.267 (0.133, 0.500)		
25–29.9	22	15.9	0.267 (0.167, 0.458)		
≥30	54	39.1	0.333 (0.100, 0.658)		
Educational				4.516	0.211
Secondary school and below	14	10.1	0.333 (0.167, 0.517)		
High school/junior college	18	13.1	0.283 (0.092, 0.608)		
College/Undergraduate	94	68.1	0.267 (0.100, 0.467)		
Master’s degree or higher	12	8.7	0.550 (0.317, 0.700)		
Residence				1.614	0.446
City	105	76.1	0.267 (0.100, 0.567)		
Town	23	16.7	0.367 (0.233, 0.500)		
Countryside	10	7.2	0.350 (0.250, 0.675)		
Employment status				−0.774	0.439
Employed	123	89.1	0.267 (0.133, 0.567)		
Unemployed	15	10.9	0.333 (0.000, 0.500)		
Monthly per capita household income (yuan)				0.931	0.628
≤3,000	18	13.0	0.283 (0.100, 0.442)		
3,000–6,000	57	41.3	0.267 (0.117, 0.517)		
≥6,000	63	45.7	0.333 (0.133, 0.600)		
Marital relationships				3.163	0.206
Cohesion	124	89.9	0.267 (0.108, 0.525)		
General	6	4.3	0.533 (0.200, 1.033)		
Occasional quarrel	8	5.8	0.367 (0.233, 0.900)		
Intergenerational relations (with mothers-in-law)				10.903	0.004
Cohesion	96	69.6	0.250 (0.100, 0.400)		
General	36	26.1	0.450 (0.267, 0.675)		
Occasional quarrel	6	4.3	0.517 (0.225, 1.200)		
Sleep situation				5.243	0.073
Regularly	101	73.2	0.267 (0.117, 0.433)		
Irregularly	24	17.4	0.467 (0.200, 0.817)		
Occasional insomnia	13	9.4	0.500 (0.150, 0.817)		
Parity				−1.623	0.105
Primiparity	89	64.5	0.333 (0.150, 0.583)		
Multiparous	49	35.5	0.233 (0.100, 0.433)		
Abortion experience				−0.846	0.398
Yes	78	56.5	0.300 (0.158, 0.567)		
No	60	43.5	0.267 (0.100, 0.533)		
Pregnancy intention				−2.717	0.007
Planned pregnancy	94	68.1	0.300 (0.192, 0.700)		
Unplanned pregnancy	44	31.9	0.200 (0.075, 0.400)		
Desired mode of delivery				−5.554	0.000
Vaginal delivery	79	57.2	0.200 (0.067, 0.333)		
Cesarean delivery	59	42.8	0.500 (0.267, 0.933)		

### 3.2 Univariate analysis of pregnancy stress

There were statistically significant differences in pregnancy stress among high-risk preeclampsia women, with intergenerational relations showing the strongest association (*p* = 0.004), followed by pregnancy intention (*p* = 0.007) and desired mode of delivery (*p* < 0.001). But age, BMI, education level, residence, employment status, monthly per capita household income, parity, and abortion experience did not differ statistically significantly (*p* > 0.05).

### 3.3 Scores for pregnant stress, anxiety, depression, and self-management

Stress, anxiety, depression, and self-management capacity scores during pregnancy took the form of 0.283 (0.133, 0.542), 38.750 (32.500, 45.000), 41.250 (33.750, 50.000), and 43.000 (35.000, 53.250). Of pregnant women at high risk for preeclampsia, our results showed that 8.7% had moderate pregnancy stress and 78.3% had mild pregnancy stress. Some pregnant women may feel depressed and anxious to some extent. In Table 2, the various distribution levels of their scores were displayed.

**Table 2:** Multiple regression results with pregnancy stress as dependent variables (*N* = 138).

Variable	Level	Case (*n*)	Percentage (%)	Score, M (P_25_, P_75_)
PSRS				0.283 (0.133, 0.542)
	Null	18	13.0	
	Mild	108	78.3	
	Moderate	12	8.7	
	Severe	0	0.0	
SAS				38.750 (32.500, 45.000)
	Null	127	92.0	
	Mild	9	6.5	
	Moderate	2	1.5	
	Severe	0	0.0	
SDS				41.250 (33.750, 50.000)
	Null	102	73.9	
	Mild	19	13.8	
	Moderate	15	10.9	
	Severe	2	1.4	
SQHMP				43.000 (35.000, 53.250)

PSRS, Pregnancy Stress Rating Scale; SAS, Self-rating anxiety scale; SDS, Self-rating depression scale; SQHMP, Self-rating Questionnaire of Healthcare Management for Pregnancy.

### 3.4 Correlations between pregnancy stress, anxiety, depression, and self-management capacity during pregnancy

Table 3 reveals that pregnancy stress was positively correlated with anxiety, depression, and self-management capacity (r = 0.465, *p* < 0.001), (r = 0.437, *p* < 0.001), (r = 0.585, *p* < 0.001). Anxiety was positively associated with depression (r = 0.754, *p* < 0.001), and with self-management capacity (r = 0.422, *p* < 0.001). Additionally, self-management capacity and depression were positively correlated (r = 0.400, *p* < 0.001).

**Table 3:** Correlations among pregnancy stress, anxiety, depression, and self-management capacity (*N* = 138).

Variables	PSRS	SAS	SDS	SQHMP
PSRS	1	–	–	–
SAS	0.465***	1	–	–
SDS	0.437***	0.754***	1	–
SQHMP	0.585***	0.422***	0.400***	1

***p < 0.001; PSRS, Pregnancy Stress Rating Scale; SAS, Self-rating anxiety scale; SDS, Self-rating depression scale; SQHMP, Self-rating Questionnaire of Healthcare Management for Pregnancy.

### 3.5 Regression analysis

Significant variables were entered into the regression equation model after conducting univariate analysis and Pearson correlation analysis. As shown in Table 4, the desired mode of delivery, anxiety, and self-management capacity were significantly associated with pregnancy stress. Specifically, the regression coefficient for desired mode of delivery was 0.206 (SE = 0.049, *t* = 4.168, *p* < 0.001); the SAS were 0.011 (SE = 0.005, *t* = 2.338, *p* < 0.05); and the regression coefficient for the SQHMP was 0.010 (SE = 0.002, *t* = 4.745, *p* < 0.001). The results indicated all above three variables had a significant positive impact on pregnancy stress. In contrast, the regression coefficients for intergenerational relationships (B = –0.040, *p* = –0.385), planned pregnancies (B = –0.036, *p* = 0.485), and SDS (B = 0.003, *p* = 0.404) did not reach significance levels. The overall regression model explains 47.7% of the variance in pregnancy stress (R^2^ = 0.477), After adjusting for the number of predictors, the model accounts for 45.3% of the variance (adjusted R^2^ = 0.453), This indicates that nearly half of the variability in pregnancy stress can be explained by the combination of intergenerational relations, pregnancy planning, desired mode of delivery, anxiety, depression, and self-management capacity. The results of the analysis were statistically significant (R^2^ = 0.477, *F* = 19.88, *p* < 0.001).

**Table 4:** Descriptive statistics of pregnancy stress, anxiety, depression and self-management ratings (*N* = 138).

Scales	B	SE	Beta	*t*	*P*	95% confidence interval	
						Lower limit	Upper limit
Intergenerational relations	−0.040	0.045	−0.062	−0.872	0.385	−0.129	0.050
Planned pregnancies	−0.036	0.051	−0.047	−0.700	0.485	−0.137	0.065
Desired mode of delivery	0.206	0.049	0.287	4.168	0.000	0.108	0.303
SAS	0.011	0.005	0.237	2.338	0.021	0.002	0.021
SDS	0.003	0.003	0.083	0.836	0.404	−0.004	0.009
SQHMP	0.010	0.002	0.340	4.745	0.000	0.006	0.015

*R*^2^ = 0.477, adjusted *R*^2^ = 0.453, *F* = 19.884, *p* < 0.001. B, non-standardized estimate; SE, standard error; Beta, standardized estimate; SAS, Self-rating anxiety scale; SDS, Self-rating depression scale; SQHMP, Self-rating Questionnaire of Healthcare Management for Pregnancy.

## 4 Discussion

Our study focused on measuring pregnancy stress levels in individuals at high risk of preeclampsia, analyzing the factors that affect pregnancy stress, and exploring the relationship between them. The findings prove that there is a widespread sense of pregnancy stress among those who are at risk for preeclampsia. Pregnancy stress was substantially accompanied by feeling anxious, depressed, and self-management capacity for pregnant women. Importantly, our regression analysis revealed three key predictors of pregnancy stress: desired mode of delivery, anxiety levels, and self-management capacity, which collectively explained 45.3% of the variance in pregnancy stress after adjustment.

### 4.1 Current status of pregnancy stress in people at high risk of preeclampsia

The findings of this study showed that 78.3% of pregnant women at high risk of preeclampsia had mild pregnancy stress and 8.7% had moderate pregnancy stress, similar to the research findings of Na et al. (2022). Unexpectedly, pregnant women with preeclampsia risk factors did not experience greater levels of pregnancy stress. This could be explained by a lack of awareness among this population about preeclampsia and its risk factors, as well as bias toward potential outcomes caused by preeclampsia (Püschl et al., 2023). Frawley et al. (2020) found that 67.5% of pregnant women were uncertain about preeclampsia before pregnancy. This made us wonder if educating this demographic about preeclampsia prevention could actually make them more stressed and depressed during pregnancy. Further intervention studies are required in the future to elucidate this.

### 4.2 Factors that affect pregnancy stress in individuals who are at high risk of preeclampsia

An important element affecting the level of pregnancy stress in the pregnant women at high-risk preeclampsia is intergenerational relationships (with mother-in-law). The mother-in-law-daughter-in-law connection, a deeply embedded subject in Chinese culture, becomes more sensitive and delicate during the perinatal time (Qi et al., 2022). Pregnant women who had a subjective perception of “cohesion” on measures of intergenerational relationships with their mothers-in-law exhibited less pregnancy stress, as we found. Insufficient social support, especially insufficient interactions between generations, is a risk factor for prenatal mental health issues (Bedaso et al., 2021). In some patriarchal societies, such as India, many Middle Eastern, African and East Asian countries, women tend to live with their husbands’ families once married. So, it is inevitable that the relationship between mothers-in-law and daughters-in-law is deeply intertwined (Varghese and Roy, 2019). According to Riem et al. (2024) study, the mental health of pregnant women depends on the support provided by their husband’s parents and the quality of intergenerational relationships. Mrayan et al. (2016) said that the social support offered by traditional pregnancy notions could not always be good for new moms’ emotional health because of the tension between traditional and modern norms. Therefore, to balance family support and encourage a positive mindset concerning pregnancy and childbirth, the health care system must provide more assistance in coordinating prenatal education with the elder generation.

Another element that influences pregnant stress in those at high risk for preeclampsia is pregnancy intention. Interestingly, compared to women with unplanned pregnancies, those with planned pregnancies suffered higher levels of pregnancy stress. This finding is consistent with the results of the Rashidi et al. (2025) study. The reason for this is that, on the one hand, women with unplanned pregnancies often choose to terminate their pregnancies early in the pregnancy and are not included in the follow-up; on the other hand, women with planned pregnancies may instead suffer from greater psychological stress and anxiety due to the pursuit of the idealized expectation of becoming a “perfect mother.” Contrary to what we found, the Maxson and Miranda (2011) study found that women who had unplanned or untimely pregnancies had higher levels of depression, anxiety, and perceived stress. This may reflect that we included women with assisted reproductive pregnancies in our high-risk group for preeclampsia. Pregnancy stress was reported to be significantly higher in women who had received assisted reproduction than in those who had been naturally conceived. The major causes of this stress include worries about how the pregnancy would turn out, the mental and physical strain of the medical procedure, and the pressure from society (Öztürk et al., 2021). This was also confirmed by a cross-sectional survey of pregnancy stress between naturally conceived women and women who underwent assisted reproduction techniques, conducted by our scholar Xuehua Cao et al. (2011). Therefore, the inclusion of this population had the potential to confound the results. The further studies should be more cautious about investigating women undergoing assisted reproduction.

A particular factor that influences pregnancy stress in those at high risk for preeclampsia was the desired mode of delivery. Our research findings, similar to those reported by Luyan Liu (2009), demonstrated that pregnant women who wished for a caesarean section experienced higher levels of pregnancy stress compared to those who expected natural childbirth. A study by Xihong Zhou (2011) found that prenatal anxiety affected a woman’s choice of delivery method. Concern regarding the fetus and fear of labor pains have made psychological issues the primary cause of the rise in undiagnosed cesarean procedures. To lessen mothers’ negative feelings and lower the rate of cesarean sections, obstetricians should educate them about scientific health care and offer them comprehensive psychosocial assistance.

### 4.3 Relationship between pregnancy stress and anxiety and depression

Depression and anxiety were highly co-morbid during pregnancy, as evidenced by our study of high-risk preeclampsia patients. In a similar vein, Lee et al. (2021) confirmed a persistent association between anxiety and depression on women during pregnancy. A substantial number of studies have indicated that adverse emotional states during pregnancy can alter the immune system and physiology of the mother, such as vasoconstriction of the placenta and other small veins and arteries throughout the body, progressively leading to an increase in blood pressure and peripheral resistance (Traylor et al., 2020). According to the past study, women who experienced more distress in their first trimester were more likely to develop preeclampsia in their second trimester (Giurgescu et al., 2015; Kurki et al., 2000). Notably, a survey conducted by Dan Li et al. (2013) across pregnant women observed that unfavorable psychological states frequently resulted in inflated perceptions and emotions regarding life events, which might raise pregnancy stress levels. As a result, it is imperative that medical professionals examine pregnant patients at risk of preeclampsia for mental health issues and offer extra effective psychological support in order to relieve pregnancy stress.

### 4.4 Relationship between pregnancy stress and ability for self-management

Our research showed that self-management capacity had a positive correlation with pregnancy stress experienced. This finding suggested that moderate pregnancy stress motivated self-management behaviors to some extent, or that pregnant women with high self-management capacity were more inclined to perceive and report pregnancy stress. In a cross-sectional study of 440 pregnant women during the 2019 novel coronavirus (COVID-19) pandemic, maternal self-care was associated with COVID-19 disease perceived severity was positively associated. This result is similar to our findings, suggesting that pregnant women with high self-care are more able to perceive and report associated stressors and threats (Khazaeian et al., 2022). Similarly, another study on the similar relationship between general self-efficacy and pregnancy stress in women with preterm labor also indicated that high levels of self-efficacy may amplify pregnancy stress (Cho and Kim, 2024). However, the specific causal relationship between pregnancy stress and self-management ability needs to be explored in future prospective intervention studies. We also focused on pregnant women at high risk of preeclampsia but do not experience pregnancy stress, who appeared to lack the ability to manage their pregnancies. Interestingly, a lower incidence of preeclampsia was observed in pregnant women with enhanced self-management capacity, as evidenced by the findings of the Alnuaimi et al. (2020) study.

Given that psychosocial stress, including that associated with pregnancy, anxiety, depression, and self-management capacity, is to some extent an external factor, it can often be mitigated through appropriate interventions (Vollebregt et al., 2008). It is essential to gain insight into the correlation between adverse emotional states, such as those related to pregnancy, especially related to high-risk pregnancies with preeclampsia, and self-management capacity. And for those at high risk for preeclampsia, it ought to be that future clinical practice incorporate a quick evaluation of pregnancy stress in early prenatal care. Furthermore, pregnant women with high risk of preeclampsia but without pregnancy stress should also pay extra attention to self-management. Proactive prenatal care can help alleviate adverse pregnancy emotions and reduce stress in pregnant women at high risk of preeclampsia, thereby lowering their probability of developing preeclampsia and achieving satisfactory pregnancy outcomes.

## 5 Limitations

Several limitations must be acknowledged in this investigation. Firstly, the research instruments we utilized were four self-reported structured questionnaires that were subjective in nature. Secondly, limited sample size of the study does not permit a definitive conclusion regarding the efficacy of self-management capacity in a population afflicted with moderate-to-severe pregnancy stress. Our study investigated only one tertiary hospital in Wuxi, Jiangsu Province. A large-scale study of this population would be beneficial in elucidating the capacity to self-management across a spectrum of pregnancy stress levels. The future research multicenter studies should be conducted in areas or institutions that are diverse in terms of ethnocultural, socioeconomic background, geographic location, and prenatal care utilization. The generalizability of the findings for replication can be improved by expanding the sample and multicenter studies. While the current model was able to explain 47.7% of the variance in pregnancy stress, other important influences did exist that were not included. For example, social support. Future studies still need to incorporate more factors to create a more comprehensive model. Finally, the cross-sectional design only allows demonstrating an association among pregnancy stress, anxiety, depression, and the capacity to self-management in individuals at high risk of preeclampsia. And it is impossible to infer causality among these factors. In future longitudinal studies, a randomized controlled trial could be further conducted in a population at high risk for preeclampsia to observe whether pregnancy stress has a causal effect on anxiety, depression, and self-management ability. The research will also be included more objective indicators, such as variations in stress biomarkers like cortisol and catecholamines, as well as the prevalence of preeclampsia and complications, except subjective questionnaires.

## 6 Conclusion

In conclusion, it can be stated that stress related to pregnancy is a common occurrence among those at risk of developing preeclampsia. Pregnancy stress was found to be independently predicted by the mother-in-law relationship, pregnancy intention, preferred delivery method, anxiety, and capacity for self-management. Therefore, healthcare professionals can combine these predictors in their clinical work to quantify and assess the emotional health status of pregnant women at high risk of preeclampsia, and develop screening guidelines for pregnancy stress in this group. Our study also revealed a robust correlation among anxiety, depression, and the capacity to self-manage and cope with pregnancy stress in individuals at high risk for preeclampsia. As such, pregnant women who are screened for pregnancy stress and emotional distress can be given proper guidance to raise their awareness of self-management, so that they can face pregnancy and labor more comfortably.

## Data Availability

The raw data supporting the conclusions of this article will be made available by the authors, without undue reservation.

## References

[ref1] Alnuaimi K. Abuidhail J. Abuzaid H. (2020). The effects of an educational programme about preeclampsia on women's awareness: a randomised control trial. Int. Nurs. Rev. 67, 501–511. doi: 10.1111/inr.12626, PMID: 32964458

[ref2] Amaral L. M. Wallace K. Owens M. LaMarca B. (2017). Pathophysiology and current clinical Management of Preeclampsia. Curr. Hypertens. Rep. 19:61. doi: 10.1007/s11906-017-0757-7, PMID: 28689331 PMC5916784

[ref3] Bedaso A. Adams J. Peng W. B. Sibbritt D. (2021). The relationship between social support and mental health problems during pregnancy: a systematic review and meta-analysis. Reprod. Health 18:23. doi: 10.1186/s12978-021-01209-5, PMID: 34321040 PMC8320195

[ref4] Bernard N. Forest J. C. Tarabulsy G. M. Bujold M. Bouvier D. Giguère Y. (2019). Use of antidepressants and anxiolytics in early pregnancy and the risk of preeclampsia and gestational hypertension: a prospective study. BMC Pregnancy Childbirth 19:9. doi: 10.1186/s12884-019-2285-8, PMID: 31039756 PMC6492434

[ref5] Bosquet Enlow M. Petty C. R. Hacker M. R. Burris H. H. (2021). Maternal psychosocial functioning, obstetric health history, and newborn telomere length. Psychoneuroendocrinology 123:105043. doi: 10.1016/j.psyneuen.2020.105043, PMID: 33176222 PMC7732207

[ref6] Buglione-Corbett R. Deligiannidis K. M. Leung K. Zhang N. Lee M. Rosal M. C.. (2018). Expression of inflammatory markers in women with perinatal depressive symptoms. Arch. Womens Ment. Health 21, 671–679. doi: 10.1007/s00737-018-0834-1, PMID: 29603018

[ref7] Caixia Z. JunTao L. Jinsong G. (2019). Incidence and clinical risk factors of preeclampsia in China. J. Reprod. Med. 28, 336–341.

[ref8] Caiyun Z. Fengjuan J. Min-min H. Hong Z. Liya L. (2020). Progress on prenatal maternal stress among pregnant women in China. Chin. J. General Prac. 18, 1353–1357.

[ref9] Chen C. H. (2015). Revision and validation of a scale to assess pregnancy stress. J. Nurs. Res. 23, 25–32. doi: 10.1097/jnr.0000000000000047, PMID: 25226045

[ref10] Cho H. J. Kim J. I. (2024). Moderating effect of general self-efficacy on the relationship between pregnancy stress, daily hassles stress, and preterm birth risk in women experiencing preterm labor: a cross-sectional study. J. Korean Acad. Nurs. 54, 329–339. doi: 10.4040/jkan.24008, PMID: 39248420

[ref11] Dan Li X. X. Liu J. Ping W. (2013). The relationship between life event and pregnancy stress: the mediating effect of mental health and the moderating effect of husband support. J. Psychol. Sci. 36, 876–883.

[ref12] Delagneau G. Twilhaar E. S. Testa R. van Veen S. Anderson P. (2023). Association between prenatal maternal anxiety and/or stress and offspring's cognitive functioning: a meta-analysis. Child Dev. 94, 779–801. doi: 10.1111/cdev.13885, PMID: 36582056 PMC10952806

[ref13] Dimitriadis E. Rolnik D. L. Zhou W. Estrada-Gutierrez G. Koga K. Francisco R. P. V.. (2023). Pre-eclampsia. Nat. Rev. Dis. Primers 9:22. doi: 10.1038/s41572-023-00417-6, PMID: 36797292

[ref14] Endeshaw M. Abebe F. Bedimo M. Asart A. (2015). Diet and pre-eclampsia: a prospective multicentre case-control study in Ethiopia. Midwifery 31, 617–624. doi: 10.1016/j.midw.2015.03.003, PMID: 25862389

[ref16] Frawley N. East C. Brennecke S. (2020). Women's experiences of preeclampsia: a prospective survey of preeclamptic women at a single tertiary Centre. J. Obstet. Gynaecol. 40, 65–69. doi: 10.1080/01443615.2019.1615040, PMID: 31455184

[ref17] Freiberger A. Beckmann J. Freilinger S. Kaemmerer H. Huber M. Nagdyman N.. (2022). Psychosocial well-being in postpartum women with congenital heart disease. Cardiovisc. Diagn. Ther. 12, 389–399. doi: 10.21037/cdt-22-213, PMID: 36033219 PMC9412213

[ref18] Gao Y. Tang X. Deng R. Liu J. Zhong X. (2023). Latent trajectories and risk factors of prenatal stress, anxiety, and depression in southwestern China-a longitudinal study. Int. J. Environ. Res. Public Health 20:818. doi: 10.3390/ijerph20053818, PMID: 36900833 PMC10001100

[ref19] Gatford K. L. Andraweera P. H. Roberts C. T. Care A. S. (2020). Animal models of preeclampsia causes, consequences, and interventions. Hypertension 75, 1363–1381. doi: 10.1161/HYPERTENSIONAHA.119.14598, PMID: 32248704

[ref20] Giurgescu C. Sanguanklin N. Engeland C. G. White-Traut R. C. Park C. Mathews H. L.. (2015). Relationships among psychosocial factors, biomarkers, preeclampsia, and preterm birth in African American women: a pilot. ANR. 28, e1–e6. doi: 10.1016/j.apnr.2014.09.002, PMID: 25282477

[ref21] Hang Y. Zhihong C. Lijuan Z. (2017). Mediating effect of self-efficacy on association between pregnancy pressure and antenatal depression. Military Nursing. 34, 21–24.

[ref22] Heberlein E. C. Picklesimer A. H. Billings D. L. Covington-Kolb S. Farber N. Frongillo E. A. (2016). The comparative effects of group prenatal care on psychosocial outcomes. Arch. Womens Ment. Health 19, 259–269. doi: 10.1007/s00737-015-0564-6, PMID: 26260037

[ref23] Ives C. W. Sinkey R. Rajapreyar I. Tita A. T. N. Oparil S. (2020). Preeclampsia-pathophysiology and clinical presentations *JACC* state-of-the-art review. J. Am. Coll. Cardiol. 76, 1690–1702. doi: 10.1016/j.jacc.2020.08.014, PMID: 33004135

[ref24] Jinzhi Li F. T. (2011). Study of self-healthcare management level and influencing factors in pregnant women. Chin. J. Prev. Med. 45, 756–757.

[ref25] Khazaeian S. Khazaeian S. Fathnezhad-Kazemi A. (2022). Association between awareness, perceived severity, and behavioral control of COVID -19 with self-Care and anxiety in pregnancy: a cross-sectional study. Women Health 62, 55–67. doi: 10.1080/03630242.2021.2014020, PMID: 34933664

[ref26] Krishnamurti T. Davis A. L. Simhan H. N. (2019). Worrying yourself sick? Association between pre-eclampsia onset and health-related worry in pregnancy. Pregn. Hypertension Int. J. Womens Cardiovascular Health. 18, 55–57. doi: 10.1016/j.preghy.2019.09.003, PMID: 31525710 PMC11191549

[ref27] Kurki T. Hiilesmaa V. Raitasalo R. Mattila H. Ylikorkala O. (2000). Depression and anxiety in early pregnancy and risk for preeclampsia. Obstet. Gynecol. 95, 487–490, PMID: 10725477 10.1016/s0029-7844(99)00602-x

[ref28] László K. D. Liu X. Q. Svensson T. Wikström A. K. Li J. Olsen J.. (2013). Psychosocial stress related to the loss of a close relative the year before or during pregnancy and risk of preeclampsia. Hypertension 62, 183–189. doi: 10.1161/HYPERTENSIONAHA.111.00550, PMID: 23608651

[ref29] Lee H. Kim K. E. Kim M. Y. Park C. G. Han J. Y. Choi E. J. (2021). Trajectories of depressive symptoms and anxiety during pregnancy and associations with pregnancy stress. Int. J. Environ. Res. Public Health 18:12. doi: 10.3390/ijerph18052733, PMID: 33800371 PMC7967460

[ref30] Luyan Liu J. L. (2009). Stresses in Primiparas and their husbands during pregnancy and its related factors. J. Nurs. Sci. 24, 39–41.

[ref31] Maxson P. Miranda M. L. (2011). Pregnancy intention, demographic differences, and psychosocial health. J. Womens Health. 20, 1215–1223. doi: 10.1089/jwh.2010.2379, PMID: 21671765

[ref32] Moulaei K. Bahaadinbeigy K. Ghaffaripour Z. Ghaemi M. M. (2021). The design and evaluation of a Mobile based application to facilitate self-care for pregnant women with preeclampsia during COVID-19 prevalence. J. Biomed. Phys. Eng. 11, 551–560. doi: 10.31661/jbpe.v0i0.2103-1294, PMID: 34458202 PMC8385215

[ref33] Mrayan L. Cornish F. Dhungana N. Parfitt B. (2016). Transition to parenthood during the transition to modernity in Jordan: new parents' views on family and healthcare support systems. Appl. Nurs. Res. 32, 139–143. doi: 10.1016/j.apnr.2016.07.002, PMID: 27969017

[ref34] Na O. Y. Jie W. Jina L. Ping Y. E. Jiayou L. U. O. (2022). Relationship between pregnancy stress and anxiety and depression in early pregnancy. Chin. J. Clin. Psych. 30, 968–972.

[ref35] Ngene N. C. Moodley J. (2024). Preventing maternal morbidity and mortality from preeclampsia and eclampsia particularly in low- and middle-income countries. Best Pract. Res. Clin. Obstet. Gynaecol. 94:102473. doi: 10.1016/j.bpobgyn.2024.102473, PMID: 38513504

[ref36] Niela-Vilen H. Ekholm E. Sarhaddi F. Azimi I. Rahmani A. M. Liljeberg P.. (2023). Comparing prenatal and postpartum stress among women with previous adverse pregnancy outcomes and normal obstetric histories: a longitudinal cohort study. Sexual Rep. Healthcare Off. J.the Swedish Assoc. Midwives. 35:100820. doi: 10.1016/j.srhc.2023.100820, PMID: 36774741

[ref37] Öztürk R. Herbell K. Morton J. Bloom T. (2021). "The worst time of my life": treatment-related stress and unmet needs of women living with infertility. J. Community Psychol. 49, 1121–1133. doi: 10.1002/jcop.22527, PMID: 33616236 PMC8324009

[ref38] Phipps E. A. Thadhani R. Benzing T. Karumanchi S. A. (2019). Pre-eclampsia: pathogenesis, novel diagnostics and therapies. Nat. Rev. Nephrol. 15, 275–289. doi: 10.1038/s41581-019-0119-6, PMID: 30792480 PMC6472952

[ref39] Püschl I. C. de Wolff M. G. Broberg L. Macklon N. Hegaard H. K. (2023). Pregnant women's attitudes to and experiences with a smartphone-based self-test for prediction of pre-eclampsia: a qualitative descriptive study. BMJ Open 13:e065575. doi: 10.1136/bmjopen-2022-065575, PMID: 37221028 PMC10230945

[ref40] Qi W. J. Liu Y. Lv H. C. Ge J. Meng Y. C. Zhao N.. (2022). Effects of family relationship and social support on the mental health of Chinese postpartum women. BMC Pregnancy Childbirth 22:10. 35078423 10.1186/s12884-022-04392-w PMC8787939

[ref41] Rahnemaei F. A. Fashami M. A. Abdi F. Abbasi M. (2020). Factors effective in the prevention of preeclampsia:a systematic review. Taiwan. J. Obstet. Gynecol. 59, 173–182. doi: 10.1016/j.tjog.2020.01.002, PMID: 32127134

[ref42] Rana S. Lemoine E. Granger J. P. Karumanchi S. A. (2020). Preeclampsia: pathophysiology, challenges, and perspectives. Circ. Res. 126, E8–E. doi: 10.1161/CIRCRESAHA.118.313276 30920918

[ref43] Rashidi F. Ghahremani F. Mahmoodi Z. Doulabi M. A. (2025). The role of social determinants of health in woman's intention to pregnancy: a model with the mediation of social support. BMC Public Health 25:1062. doi: 10.1186/s12889-025-22223-3, PMID: 40108586 PMC11921526

[ref44] Riem M. M. E. Perrykkad K. Watson S. J. Wynter K. Ijzendoorn M. H. V. Galbally M. (2024). The role of lack of grandparental support in perinatal depression. J. Affect. Disord. 360, 198–205. doi: 10.1016/j.jad.2024.05.104, PMID: 38788855

[ref45] Shay M. MacKinnon A. L. Metcalfe A. Giesbrecht G. Campbell T. Nerenberg K.. (2020). Depressed mood and anxiety as risk factors for hypertensive disorders of pregnancy: a systematic review and meta-analysis. Psychol. Med. 50, 2128–2140. doi: 10.1017/S0033291720003062, PMID: 32912348

[ref46] Shuxiu L. (2021). Research on child-rearing dilemma and its influence on fertility intention in the City Group of Yangtze River Delta region. Youth Expl. 3, 88–98.

[ref47] Smith C. A. Tuson A. Thornton C. Dahlen H. G. (2020). The safety and effectiveness of mind body interventions for women with pregnancy induced hypertension and or preeclampsia: a systematic review and meta-analysis. Complement. Ther. Med. 52:102469. doi: 10.1016/j.ctim.2020.102469, PMID: 32951719

[ref48] Tang X. Lu Z. Hu D. H. Zhong X. N. (2019). Influencing factors for prenatal stress, anxiety and depression in early pregnancy among women in Chongqing, China. J. Affect Disord. 253, 292–302. doi: 10.1016/j.jad.2019.05.003, PMID: 31077972

[ref49] Traylor C. S. Johnson J. D. Kimmel M. C. Manuck T. A. (2020). Effects of psychological stress on adverse pregnancy outcomes and nonpharmacologic approaches for reduction: an expert review. Amer. J. Obstet. Gynecol. MFM. 2:100229. doi: 10.1016/j.ajogmf.2020.100229, PMID: 32995736 PMC7513755

[ref50] Tuxunnjiang X. B. D. Wumaier G. L. J. N. T. Ting J. (2022). The relationship between pregnancy stress and prenatal anxiety andsocial support in pregnant women. Modern. Prev. Med. 49, 2178–2183.

[ref51] Varghese R. Roy M. (2019). Coresidence with mother-in-law and maternal anemia in rural India. Soc. Sci. Med. 226, 37–46. doi: 10.1016/j.socscimed.2019.02.027, PMID: 30836297

[ref52] Vollebregt K. C. van der Wal M. F. Wolf H. Vrijkotte T. G. M. Boer K. Bonsel G. J. (2008). Is psychosocial stress in first ongoing pregnancies associated with pre-eclampsia and gestational hypertension? Bjog-Int. J. Obstetrics Gynaecol. 115, 607–615. doi: 10.1111/j.1471-0528.2008.01665.x, PMID: 18333942

[ref53] Wang Y. C. Gu J. Zhang F. Xu X. J. (2023). The mediating role of social support and resilience between self-efficacy and prenatal stress: a mediational analysis. BMC Pregnancy Childbirth 23:10. doi: 10.1186/s12884-023-06184-2, PMID: 38104088 PMC10724952

[ref54] Xiao Q. L. Jun Y. C. Hong H. L. (2024). Investigation on the current status of standard pregnancy health Care and self-management behavior of rural women. Chin. Gen. Pract. 22, 1–7.

[ref55] Xihong Zhou L. L. (2011). Prenatal anxiety and its influence on delivery outcome. J. Central South Univ. 36, 803–808. 10.3969/j.issn.1672-7347.2011.08.020 21937812

[ref56] Xuehua Cao L. M. Zhang X. Qun L. V. Zhou L. (2011). Stress and its causes of pregnant women undergoing assisted reproductive technology. Clin. Med. 8, 119–120.

[ref57] Yao Tang W. G. Li X. (2022). Interpretation of the 2021 ISSHP clinical guidelines for disorders of hypertension in pregnancy. Journal of practical. Obstet. Gynecol. 38, 832–838.

[ref58] Yu Y. X. Zhang S. C. Wang G. Y. Hong X. M. Mallow E. B. Walker S. O.. (2013). The combined association of psychosocial stress and chronic hypertension with preeclampsia. Am. J. Obstet. Gynecol. 209:12. doi: 10.1016/j.ajog.2013.07.003 PMC3825759 23850528

[ref59] Zhou Y. Huang J. G. Baker P. N. Liao B. Z. Yu X. Y. (2022). The prevalence and associated factors of prenatal depression and anxiety in twin pregnancy: a cross-sectional study in Chongqing, China. BMC Pregnancy Childbirth 22:10. doi: 10.1186/s12884-022-05203-y, PMID: 36435754 PMC9701401

[ref60] Zung W. W. (1971). A rating instrument for anxiety disorders. Psychosomatics 12, 371–379. doi: 10.1016/S0033-3182(71)71479-0 5172928

