# Supplementary material for: Pregnancy stress in women at high risk of preeclampsia with their anxiety, depression, self-management capacity: a cross-sectional study
Source: Front Psychol. 2025 May 21;16:1537858. doi: 10.3389/fpsyg.2025.1537858 (PMC12133748; doi:10.3389/fpsyg.2025.1537858)
Supplement: Supplementary file 1 [file Supplementary_file_1.docx]

**Self-rating depression scale (SDS)**

Please read each entry carefully and then tick the corresponding box according to how you have actually felt in the last week.

| Assessment items | None or few  (<1 day/ week) | sometimes  (1－2 days/ week) | Most of the time  (3－4 days/ week) | Vast majority of the time  (5－7 days/ week) |
| --- | --- | --- | --- | --- |
| 1. I feel sullen and depressed |  |  |  |  |
| 2. I think the morning is the best part of the day |  |  |  |  |
| 3. I cry out in bursts or feel like crying |  |  |  |  |
| 4. I don't sleep well at night |  |  |  |  |
| 1. I ate as much as usual |  |  |  |  |
| 6. I feel as pleasant as ever when I'm intimate with the opposite sex. |  |  |  |  |
| 7. I realized I was losing weight. |  |  |  |  |
| 8. I suffer from constipation |  |  |  |  |
| 9. My heart is beating faster than usual |  |  |  |  |
| 10. I'm tired for no reason. |  |  |  |  |
| 11. My mind is as clear as usual |  |  |  |  |
| 12. I don't find it difficult to do things regularly |  |  |  |  |
| 13. I feel uneasy and unsettled |  |  |  |  |
| 14. I have hope for the future |  |  |  |  |
| 15. I get angry and agitated more easily than usual |  |  |  |  |
| 16. I find it easy to make decisions. |  |  |  |  |
| 17. I feel like I'm a useful person and someone needs me |  |  |  |  |
| 18. I've had an interesting life. |  |  |  |  |
| 19. I think people would be better off if I died. |  |  |  |  |
| 20. I'm still interested in the same things I'm usually interested in. |  |  |  |  |
