# Supplementary material for: Pregnancy stress in women at high risk of preeclampsia with their anxiety, depression, self-management capacity: a cross-sectional study
Source: Front Psychol. 2025 May 21;16:1537858. doi: 10.3389/fpsyg.2025.1537858 (PMC12133748; doi:10.3389/fpsyg.2025.1537858)
Supplement: Supplementary file 2 [file Supplementary_file_2.docx]

**Self-rating Questionnaire of Healthcare Management for Pregnancy (SQHMP)**

1. **Daily intake of appropriate amount of fresh vegetables and fruits.**

(1) Do it completely (2) Do it often (3) Do it sometimes (4) Do it seldom (5) Do it not at all

**2. Supplementation of folic acid, calcium and iron during pregnancy under the guidance of a doctor.**

(1) Do it completely (2) Do it often (3) Do it sometimes (4) Do it seldom (5) Do it not at all

**3. try to eat less fried, oil only and stimulating food, such as spicy food, coffee and so on.**

(1) Do it completely (2) Do it often (3) Do it sometimes (4) Do it seldom (5) Do it not at all

**4. Insist on napping for about 1 hour every day.**

(1) Do it completely (2) Do it often (3) Do it sometimes (4) Do it seldom (5) Do it not at all

**5. Listening to music, reading novels, talking to the fetus, etc. to carry out “fetal education”.**

(1) Do it completely (2) Do it often (3) Do it sometimes (4) Do it seldom (5) Do it not at all

**6. Weigh yourself at home every 1-2 weeks and record it.**

(1) Do it completely (2) Do it often (3) Do it sometimes (4) Do it seldom (5) Do it not at all

**7. During pregnancy, sleep at night or during the day in the left lateral position.**

(1) Do it completely (2) Do it often (3) Do it sometimes (4) Do it seldom (5) Do it not at all

**8. Minimize visits to crowded places after pregnancy.**

(1) Do it completely (2) Do it often (3) Do it sometimes (4) Do it seldom (5) Do it not at all

**9. Avoid heavy physical labor and strenuous exercise.**

(1) Do it completely (2) Do it often (3) Do it sometimes (4) Do it seldom (5) Do it not at all

**10. During pregnancy, you actively avoided passive smoking environments.**

(1) Do it completely (2) Do it often (3) Do it sometimes (4) Do it seldom (5) Do it not at all

**11. Appropriate daily exercise during pregnancy, such as walking.**

(1) Do it completely (2) Do it often (3) Do it sometimes (4) Do it seldom (5) Do it not at all

**12. Avoid maintaining a certain posture for a long time, such as standing, sitting or walking.**

(1) Do it completely (2) Do it often (3) Do it sometimes (4) Do it seldom (5) Do it not at all

**13. At 28 weeks of pregnancy, count fetal movements once a day in the morning, midday, and evening.**

(1) Do it completely (2) Do it often (3) Do it sometimes (4) Do it seldom (5) Do it not at all

**14. Fetal movements lasting 1 hour each time.**

(1) Do it completely (2) Do it often (3) Do it sometimes (4) Do it seldom (5) Do it not at all

**15. Record the number of fetal movements in a timely manner after each count.**

(1) Do it completely (2) Do it often (3) Do it sometimes (4) Do it seldom (5) Do it not at all

**16. The fetus moves less or more frequently and then goes to the hospital in time.**

(1) Do it completely (2) Do it often (3) Do it sometimes (4) Do it seldom (5) Do it not at all

**17. When you feel unwell during pregnancy, such as vaginal bleeding, lumbago, pain, fever, headache, headache, etc., you will go to the hospital in time for medical treatment.**

(1) Do it completely (2) Do it often (3) Do it sometimes (4) Do it seldom (5) Do it not at all

**18. Being able to come to the hospital for prenatal checkups on time and in accordance with the doctor's instructions during pregnancy.**

(1) Do it completely (2) Do it often (3) Do it sometimes (4) Do it seldom (5) Do it not at all

**19. During prenatal checkups, take the initiative to consult doctors about the health status of the mother and child and the precautions to be taken.**

(1) Do it completely (2) Do it often (3) Do it sometimes (4) Do it seldom (5) Do it not at all

**20. Being able to use medicines rationally under the guidance of a doctor during pregnancy, such as all kinds of antibiotics.**

(1) Do it completely (2) Do it often (3) Do it sometimes (4) Do it seldom (5) Do it not at all

**21. Do ultrasound on time as prescribed by the doctor to check the development of the fetus.**

(1) Do it completely (2) Do it often (3) Do it sometimes (4) Do it seldom (5) Do it not at all

**22. Perform other tests such as blood and urine as prescribed by the doctor and consult whether the results are normal or not.**

(1) Do it completely (2) Do it often (3) Do it sometimes (4) Do it seldom (5) Do it not at all

**23. Regular participation in the “School for Pregnant Women” and exchange of experience and information with other pregnant women.**

(1) Do it completely (2) Do it often (3) Do it sometimes (4) Do it seldom (5) Do it not at all

**24. Take the initiative to obtain health care knowledge from family members, friends or other sources such as books, computers, etc.**

(1) Do it completely (2) Do it often (3) Do it sometimes (4) Do it seldom (5) Do it not at all

**25. Be able to take the initiative to self-regulate when in a troubled mood, such as talking to family members, listening to music, taking a walk, etc.**

(1) Do it completely (2) Do it often (3) Do it sometimes (4) Do it seldom (5) Do it not at all
