# Supplementary material for: Pregnancy stress in women at high risk of preeclampsia with their anxiety, depression, self-management capacity: a cross-sectional study
Source: Front Psychol. 2025 May 21;16:1537858. doi: 10.3389/fpsyg.2025.1537858 (PMC12133748; doi:10.3389/fpsyg.2025.1537858)
Supplement: Supplementary file 3 [file Supplementary_file_3.docx]

**Pregnancy Stress Rating Scale (PSRS)**

Below is a list of stressful situations you may encounter during your pregnancy, with four choices for each entry: 1, 2, 3 and 4. “1” indicates that the condition does not exist or does not cause you stress at all; a “2” indicates that the condition exists and causes you a low level of stress; a “3” indicates that it causes you a moderate level of stress; and a “4” indicates that it causes you a high level of stress. Please select an answer based on your actual situation and tick the appropriate number.

| Assessment items | None | mild | moderate | severe |
| --- | --- | --- | --- | --- |
| 1. Difficulty in preparing baby clothes | 1 | 2 | 3 | 4 |
| 2. Difficulty in finding a satisfactory nanny | 1 | 2 | 3 | 4 |
| 3. Difficulty in choosing a place to sit for the baby's first day of the month | 1 | 2 | 3 | 4 |
| 4. It's hard to name your child | 1 | 2 | 3 | 4 |
| 5. Fear that significant others will not accept the child | 1 | 2 | 3 | 4 |
| 6. Difficulty in giving the fetus a physical examination | 1 | 2 | 3 | 4 |
| 7. Fear that they will be forced to give up their jobs if they have children | 1 | 2 | 3 | 4 |
| 8. Inability to organize household chores during childbirth | 1 | 2 | 3 | 4 |
| 9. Fear of inadequate psychological support | 1 | 2 | 3 | 4 |
| 10. Difficulty in deciding how to feed the infant | 1 | 2 | 3 | 4 |
| 11. Fear that the sex of the baby is not what is expected | 1 | 2 | 3 | 4 |
| 12. Fear of affecting sex life | 1 | 2 | 3 | 4 |
| 13. Worried that the child will not be liked | 1 | 2 | 3 | 4 |
| 14. Worried about future child support | 1 | 2 | 3 | 4 |
| 15. Worried about having less free time after having children | 1 | 2 | 3 | 4 |
| 16. Concerns about the safe delivery of the fetus | 1 | 2 | 3 | 4 |
| 17. Fear of fetal abnormality | 1 | 2 | 3 | 4 |
| 18. Worried about the safety of their delivery | 1 | 2 | 3 | 4 |
| 19. Fear of premature labor | 1 | 2 | 3 | 4 |
| 20. Worry about the weight of the fetus | 1 | 2 | 3 | 4 |
| 21. Fear of possible irregularities in delivery or caesarean section | 1 | 2 | 3 | 4 |
| 22. Fear that the doctor will not arrive in time for delivery | 1 | 2 | 3 | 4 |
| 23. Fear of being in severe pain during labor | 1 | 2 | 3 | 4 |
| 24. Worried about changing their body shape | 1 | 2 | 3 | 4 |
| 25. Worry about stretch marks on your face | 1 | 2 | 3 | 4 |
| 26. Worry about getting too fat | 1 | 2 | 3 | 4 |
| 27. Worry about not being able to control your clumsy body | 1 | 2 | 3 | 4 |
| 28. Worried about not being able to take care of the baby | 1 | 2 | 3 | 4 |
| 29. Worried that having children will affect the couple's relationship | 1 | 2 | 3 | 4 |
| 30. Fear of not being able to provide good living conditions for their children | 1 | 2 | 3 | 4 |
