# Supplementary material for: Pregnancy stress in women at high risk of preeclampsia with their anxiety, depression, self-management capacity: a cross-sectional study
Source: Front Psychol. 2025 May 21;16:1537858. doi: 10.3389/fpsyg.2025.1537858 (PMC12133748; doi:10.3389/fpsyg.2025.1537858)
Supplement: Supplementary file 4 [file Supplementary_file_4.docx]

**Self-rating anxiety scale (SAS)**

Please read each entry carefully and then tick the corresponding box according to how you have actually felt in the last week.

| Assessment items | None or few  (<1 day/ week) | sometimes  (1－2 days/ week) | Most of the time  (3－4 days/ week) | Vast majority of the time  (5－7 days/ week) |
| --- | --- | --- | --- | --- |
| 1. I feel more nervous and anxious than usual |  |  |  |  |
| 2. I'm scared for no reason. |  |  |  |  |
| 3. I get upset or feel panicky easily |  |  |  |  |
| 4. I think I might be going crazy |  |  |  |  |
| 5. I think everything is fine and nothing unfortunate will happen |  |  |  |  |
| 6. My hands and feet are shaking and trembling |  |  |  |  |
| 7. I suffer from headaches, head and neck pains and back pains |  |  |  |  |
| 8. I feel easily debilitated and fatigued |  |  |  |  |
| 9. I feel calm, and it is easy to sit quietly |  |  |  |  |
| 10. I feel my heart beating fast |  |  |  |  |
| 11. I suffered from a bout of dizziness |  |  |  |  |
| 12. I have fainting episodes or feel like I'm going to faint |  |  |  |  |
| 13. It's easy for me to breathe in and out. |  |  |  |  |
| 14. numbness and tingling in my hands and feet |  |  |  |  |
| 15. I'm suffering from stomach pains and indigestion. |  |  |  |  |
| 16. I have to pee a lot. |  |  |  |  |
| 17. My hands are often dry and warm |  |  |  |  |
| 18. I'm blushing hot |  |  |  |  |
| 19. I fall asleep easily and sleep well through the night. |  |  |  |  |
| 20. I have nightmares. |  |  |  |  |
